# Magnetic Resonance Imaging Assessment of Effective Ablated Volume following High Intensity Focused Ultrasound

**DOI:** 10.1371/journal.pone.0120037

**Published:** 2015-03-18

**Authors:** Brett Z. Fite, Andrew Wong, Yu Liu, Lisa M. Mahakian, Sarah M. Tam, Olulanu Aina, Neil E. Hubbard, Alexander Borowsky, Robert D. Cardiff, Erik Dumont, Katherine W. Ferrara

**Affiliations:** 1 Department of Biomedical Engineering, University of California Davis, Davis, CA, 95616, United States of America; 2 Center for Comparative Medicine, University of California Davis, Davis, CA, 95616, United States of America; 3 Department of Pathology and Laboratory Medicine, School of Medicine, University of California Davis, Davis, CA, 95616, United States of America; 4 Image Guided Therapy, Pessac, France; Tongji University, Tenth People's Hospital of Tongji University, CHINA

## Abstract

Under magnetic resonance (MR) guidance, high intensity focused ultrasound (HIFU) is capable of precise and accurate delivery of thermal dose to tissues. Given the excellent soft tissue imaging capabilities of MRI, but the lack of data on the correlation of MRI findings to histology following HIFU, we sought to examine tumor response to HIFU ablation to determine whether there was a correlation between histological findings and common MR imaging protocols in the assessment of the extent of thermal damage. Female FVB mice (n = 34), bearing bilateral *neu* deletion tumors, were unilaterally insonated under MR guidance, with the contralateral tumor as a control. Between one and five spots (focal size 0.5 × 0.5 × 2.5 mm^3^) were insonated per tumor with each spot receiving approximately 74.2 J of acoustic energy over a period of 7 seconds. Animals were then imaged on a 7T MR scanner with several protocols. T1 weighted images (with and without gadolinium contrast) were collected in addition to a series of T2 weighted and diffusion weighted images (for later reconstruction into T2 and apparent diffusion coefficient maps), immediately following ablation and at 6, 24, and 48 hours post treatment. Animals were sacrificed at each time point and both insonated/treated and contralateral tumors removed and stained for NADH-diaphorase, caspase 3, or with hematoxylin and eosin (H&E). We found the area of non-enhancement on contrast enhanced T1 weighted imaging immediately post ablation correlated with the region of tissue receiving a thermal dose CEM43 ≥ 240 min. Moreover, while both tumor T2 and apparent diffusion coefficient values changed from pre-ablation values, contrast enhanced T1 weighted images appeared to be more senstive to changes in tissue viability following HIFU ablation.

## Introduction

Magnetic resonance guided focused ultrasound (MRgFUS) is a noninvasive method for the application of thermal and mechanical therapies. High intensity focused ultrasound (HIFU), when applied via an extracorporeal transducer, noninvasively delivers acoustic energy to body tissue with a high degree of precision; depending upon the array geometry and frequency, the precision can be on the order of a few millimeters. MRI guidance provides a high degree of precision in localizing the focal point of the ultrasound beam in addition to providing 2D or 3D temperature maps in real time for monitoring energy deposition in tissue and calculating thermal dose maps peri- and post treatment [1–5]. Furthermore, the variety of MRI protocols available for anatomical and functional imaging make MRI a natural choice for both identifying the target tissue and assessing tissue response to thermal or mechanical therapies during and after therapy.

Temperature change measurements made with the proton resonance frequency shift (PRFS) of water protons are especially suited to the control of thermal ablation due to the wide linearity of the effect, even following coagulation [6]. In addition to MRI guidance, thermal therapies can be guided by ultrasound [7], x-ray computed tomography (CT) [8], or via the use of an interstitial temperature probe. The latter, while easy to use and accurate, provides only a single spatial measurement point leaving thermal dose away from the probe uncertain, which can be especially problematic in larger tumors. Ultrasound imaging has been used to accurately monitor thermal ablation [9], but it can be difficult to quantify thermal dose at ablative temperature with current techniques [10]. X-ray CT offers high temporal and spatial resolution, but entails ionizing radiation exposure and exogenous contrast agents are also typically required.

HIFU induced thermally ablative therapies are a promising alternative to conventional surgery [11–13], and are increasingly being investigated and employed for treatment of cancers of the prostate [14], breast [15], bladder [16], liver [17] and other solid tumors [18], as well as uterine fibroids [19–21]. Moreover, ablative therapies can be combined synergistically with radiotherapy and chemotherapy [22, 23]. In contrast to hyperthermia, ablative thermal therapies typically result in temperatures of at least 50°C in the target tissue, with the intention of achieving thermal coagulation over the treated region [24]. A minimal thermal dose, defined as the cumulative equivalent minutes at 43°C (CEM_43_) of 240 min [25], is often chosen as the contour of lethal exposure for post treatment assessment, but variations in tissue response to thermal therapy can make correlation of thermal dose to histology challenging [26, 27]. Moreover, the high rate of change of temperature during ablative procedures can make accurate magnetic resonance thermometry (MRT), and thus accurate calculation of thermal dose, difficult. This is especially true on lower field clinical systems (e.g. 1.5T, 3T) where there is less available signal to noise ratio (SNR) to allocate to increase temporal resolution while maintaining spatial and thermal resolutions. Coupled with the fact that when operating above 43°C the thermal dose doubles for each temperature increase of 1°C, it is clear that having an alternative method to evaluate tissue damage (beyond thermal dose) is often desirable.

In addition to examining post treatment thermal dose contours, imaging studies are also used to estimate tissue death several days to several weeks after treatment, where contrast-enhanced T1-weighted (T1w) imaging is most often applied for imaging of tissue death. Gadolinium contrast-enhanced T1w imaging has shown great utility in assessing the extent of tissue damage following HIFU [28], and dynamic contrast enhanced MRI has also been shown to be a good predictor of metastasis free survival following a combination of hyperthermia and radiotherapy [29]. However, some studies have suggested that T1w MRI cannot predict histological results in the prostate [30]. Conversely, work in breast cancer has shown poor visualization of thermal tissue damage at early post treatment time points. However, beyond 24 hours post treatment, thermal damage was visualized by MRI and the images correlated with tissue histology [31]. Contrast enhanced MRI was evaluated as a monitoring procedure for laser induced thermotherapy and found to correlate well with tissue death [32]. More recently, multiparametric MRI was shown to yield good agreement with histological results in differentiating viable and non-viable tumor [33].

The relationship between MR imaging results and histological findings following HIFU ablation is not always straightforward. Further confounding this issue is the ability of HIFU to heat-fix cells, giving ablated tissue a similar appearance to viable tissue on hematoxylin and eosin (H&E) [34]. This can both underestimate the true extent of tissue damage and erroneously suggest differences between MR findings and actual tissue damage.

Work with radiofrequency (RF) induced thermal ablation has also suggested that MR imaging can identify irreversible thermal damage prior to its becoming evident on the standard H&E stain [35]. Thus, the use of a specialty viability stain, such as those using tetrazolium dyes, is desirable to accurately differentiate viable from non-viable tissue, especially shortly after thermal damage when heat-fixed cells remain at the damage site. Additionally, cells which receive a smaller, but still lethal thermal dose, may become pre-apoptotic [36], and thus will contribute to the ultimate volume of tissue death but will appear viable on both H&E and viability stains at early time points. Therefore, immunohistochemistry (IHC) for markers of apoptosis, such as activated caspase 3 [37], are also valuable in determining the boundary of tissue death following thermal HIFU treatments.

Therefore, in this study, we sought to examine changes in tumor tissue at several time points following HIFU ablation using T1w, contrast enhanced T1w, T2 weighted (T2w), and diffusion weighted (DW) imaging. We choose to compare contrast enhanced T1w imaging due to the ability to examine changes in tissue perfusion, DW imaging to detect changes in water diffusion due to coagulation and changes in cellular density and T2w imaging to visualize edema. Additionally, we validated the imaging studies with histological stains to detect immediate tissue death as well as apoptotic cells.

## Materials and Methods

### Ethics Statement

All animal experiments were performed under a protocol approved by the Institutional Animal Care and Use Committee (IACUC) of the University of California, Davis. The animal experiments for this study were specifically approved by the IACUC of the University of California, Davis under protocol # 15717. Anesthesia and post treatment analgesia was administered for all imaging and surgical procedures. All mice were housed in accordance with approved IACUC protocols.

### HIFU System

An MR compatible 16 element annular array (IMASONIC SAS, Voray sur l'Ognon, France) with a 3MHz center frequency and capable of 100W acoustic power (focal spot size 0.5 × 0.5 × 2.5 mm^3^) was used for the thermal HIFU treatments [38].

### Histology

Tumor samples were divided into two halves: one half was flash frozen and cut into 20 μm sections for NADH-diaphorase staining, while the other half was preserved in neutral-buffered formalin for H&E staining and IHC for activated caspase 3. NADH stained sections were brought to reach room temperature and then incubated for 15 minutes at room temperature with 50 μL of nitroblue tetrazolium media. This media contained 1.25 mL of nitroblue tetrazolium at 2.5 mg/mL in distilled water, 0.5 mL DPBS without calcium or magnesium, 0.25 mL Ringer’s solution, and 0.5 mL of α-NADH 2.5 mg/mL in distilled water, prepared immediately before use. Slides were then washed for 30 seconds each in 7 sequential concentrations of acetone in distilled water (0%, 30%, 60%, 90%, 60%, 30%, 0%). Finally, slides were washed for 30 seconds with five changes of tap water followed by one change of distilled water and were subsequently coverslipped with an aqueous mounting medium.

For H&E, a Tissue-Tek VIP autoprocessor (Sakura, Torrance, CA) was used to process tumors which were then embedded in Paraplast paraffin (melting temperature 56–60°C), sectioned to 5 μm and mounted on glass slides. Tumor sections were then stained using Mayer’s H&E to facilitate histology and morphology evaluation.

Tumor samples were also immunostained for activated caspase 3 using a rabbit anti-mouse activated caspase 3 polyclonal antibody (1:1000, Promega, Madison, WI). A goat anti-rabbit secondary antibody was used for signal amplification (Vectastain ABC Kit, Vector Labs, Burlingame, CA). The antibody complexes were detected using the DAB Peroxidase Substrate Kit (Vector Labs). Both H&E and caspase stained slides were scanned using an Aperio XT ScanScope (Aperio, Vista, CA) and digital images were accessed from the Spectrum database associated with the ScanScope and processed.

### Animal Model

The *neu* deletion (NDL) cell line is a metastatic mammary carcinoma, originating from the over-expression of the ErbB-2/neu proto-oncogene [39, 40]. The Neu protein, a type I subclass of receptor tyrosine kinases, has been linked to initiation and progression of breast cancer. This cell line yields focal mammary adenocarcinomas that evolve and metastasize to the lung with high frequency. For tumor transplantation, a bolus of cells (1 x 10^5^) was injected bilaterally into the uncleared #4 fat pads of 6 week old mice. Following transplantation, mice were palpated twice weekly to monitor tumor take, which equaled or exceeded 90%. The tumors were usually palpable within 10 to 14 d after transplantation. The tumors were allowed to grow to 5 mm to 10 mm in greatest dimension. In our experience, tumors larger than 10 mm are frequently necrotic, resulting in fewer viable tumor cells. To remove the tumors, mice were first anesthetized using Nembutal (60 mg/kg). Following tumor removal, the mice were euthanized using an overdose of Nembutal (120 mg/kg). All tumors were immediately cut into halves using a razor blade or scalpel and placed immediately into the experimental fixative or snap frozen.

### Ablation Protocol

Animals were anesthetized during all experiments. Anesthesia was induced with 3% isoflurane (in oxygen, flow 2 L/min) and maintained at 1.5%. Buprenorphine (Reckitt Benckiser Healthcare) was administered subcutaneously (0.05 mg/kg) approximately 30 minutes prior to ablation for additional analgesia.

An MR compatible small animal monitoring system (Model 1025, SA Instruments, Stony Brook, NY, USA) was used to measure respiration and core body temperature throughout the course of the experiments. Animals were warmed with a forced air system (SA Instruments, Stony Brook, NY, USA).

One tumor per animal was insonated, with the contralateral tumor as a control. Between one and five spots (focal size 0.5 × 0.5 × 2.5 mm^3^) were insonated per tumor. Each spot received an approximate acoustic dose of 74.2 J (7 seconds insonation at 10.6 W acoustic power) to ensure a minimum CEM_43_ of 240. A gradient echo sequence (FLASH; TR/TE/FA = 21 ms/4.5 ms/20°; FOV = 3.2 × 3.2 cm^2^; MTX = 64 × 64; ST = 1 mm; 2 orthogonal slices in coronal and axial orientations) was acquired during insonation to monitor the temperature within the tumor and for later generation of thermal dose maps. The temperature maps were generated in real time via the PRFS of water protons [41] within Thermoguide (S1 Video), the software controlling the HIFU system, using raw MR data streamed to it over a private network from the workstation controlling the MRI scanner. At the conclusion of the thermometry scan, thermal dose maps were generated within Thermoguide using the previously calculated temperature maps.

In the first study, animals were divided into 4 groups: 0, 6, 24 and 48 hrs following ablation (Table 1). Prior to insonation, T1w, T2w and DW image series were collected (where the T2W and DW series were required for later generation of T2 and apparent diffusion coefficient (ADC) maps, respectively). Animals were then imaged post insonation with the same MRI protocols but with the addition of a gadolinium contrast enhanced T1w scan. Animals in the 0 hr group were sacrificed following these scans while animals in other groups were imaged again at their terminal time point prior to sacrifice. Following sacrifice, both treated and untreated control tumors were removed. Of each tumor, half was used for NADH-diaphorase and half for H&E staining.

**Table 1:** Number of animals imaged via MRI at each time point post ablation.

	Pre Ablation	0hr	6hr	24hr	48hr
Study 1	30 animals	13 animals	4 animals	8 animals	5 animals
Study 2	4 animals	4 animals	4 animals	4 animals	4 animals
Total	34 animals	17 animals	8 animals	12 animals	9 animals

The primary difference between the studies is that in study 1, animals were euthanized immediately following MR imaging to obtain histology on the tumors, whereas in study 2 the group of 4 animals were imaged at 0, 6, 24, and 48 hours post ablation (they were all euthanized immediately after the 48 hour imaging session) to look at changes in imaging results in the same individuals.

In a second study (Table 1), one group of animals was insonated as described above, but all animals were successively imaged at 0, 6, 24, and 48 hours before being sacrificed at the 48 hour time point for histological analysis.

### MR Imaging

A Bruker Biospec 70/30 (7T) small animal scanner (Bruker BioSpin MRI, Ettlingen, Germany) equipped with a 154 mm internal diameter, circularly polarized coil operated in transmit-only mode; signal reception was achieved by a four channel rat brain phased array surface coil in a cross coil configuration. T2 and DW scans were performed prior to gadolinium contrast administration at all time points. Data were acquired and images were reconstructed using ParaVision 5.1 (Bruker BioSpin MRI). Parametric images were generated with either ParaVision 5.1 or MATLAB (Mathworks).

A segmented echo planar sequence (echo time (TE)/repetition time (TR) = 38.2/3000 ms, field of view (FOV) = 3.2 × 3.2 cm^2^; matrix size (MTX) = 192 × 192; slice thickness (ST)/slice interval (SI) = 1 mm/1 mm, 7 slices, 5 segments, receiver bandwidth (BW) = 238 kHz) was used to acquire a series of DWIs with varying diffusion weighting (b = 0, 100, 200, 400, 600, 800, 1000, 1250, 1500, 2000 s/mm^2^), which were reconstructed to ADC maps by fitting to a monoexponential curve within ParaVision.

T2w images were acquired (TR = 5000 ms; FOV = 3.2 × 3.2 cm^2^; MTX = 128 × 128; ST/SI = 1 mm/1 mm; 9 slices, BW = 81 kHz) were acquired with varying echo times (TE = 13, 26, 78, 130, 182, 234, 286, 338, 390 ms). T2 map were generated by fitting to a monoexponential cruve within ParaVision.

A T1w scan (TE/TR = 12.5 ms/750 ms; FOV = 3.2 × 3.2 cm^2^; MTX = 256 × 256, ST/SI = 1 mm/1 mm; 9 slices; BW = 81 kHz) was also performed both before and after administration of gadolinium contrast (0.5 μmol/g Prohance, intravenous (IV) bolus).

The non-perfused area (NPA), suggesting inhibited perfusion, was calculated as the area of non-enhancement on T1w images and compared to the area of the tumor that received a CEM_43_ ≥ 240 min, as determined from the thermal dose maps. The non-perfused volume (NPV) was calculated as the NPA for a given slice multiplied by the slice thickness for that slice, then summed over all slices containing a non-perfused area.

### Statistical Analysis

Statistical analyses were performed using Excel 11.0 (Microsoft, Seattle, WA). Data were recorded as mean ± standard deviation unless otherwise specified. A two-tailed Student's t test was used to test for significance with a P value less than 0.05 indicating a statistically significant difference.

## Results

**Fig 1:**
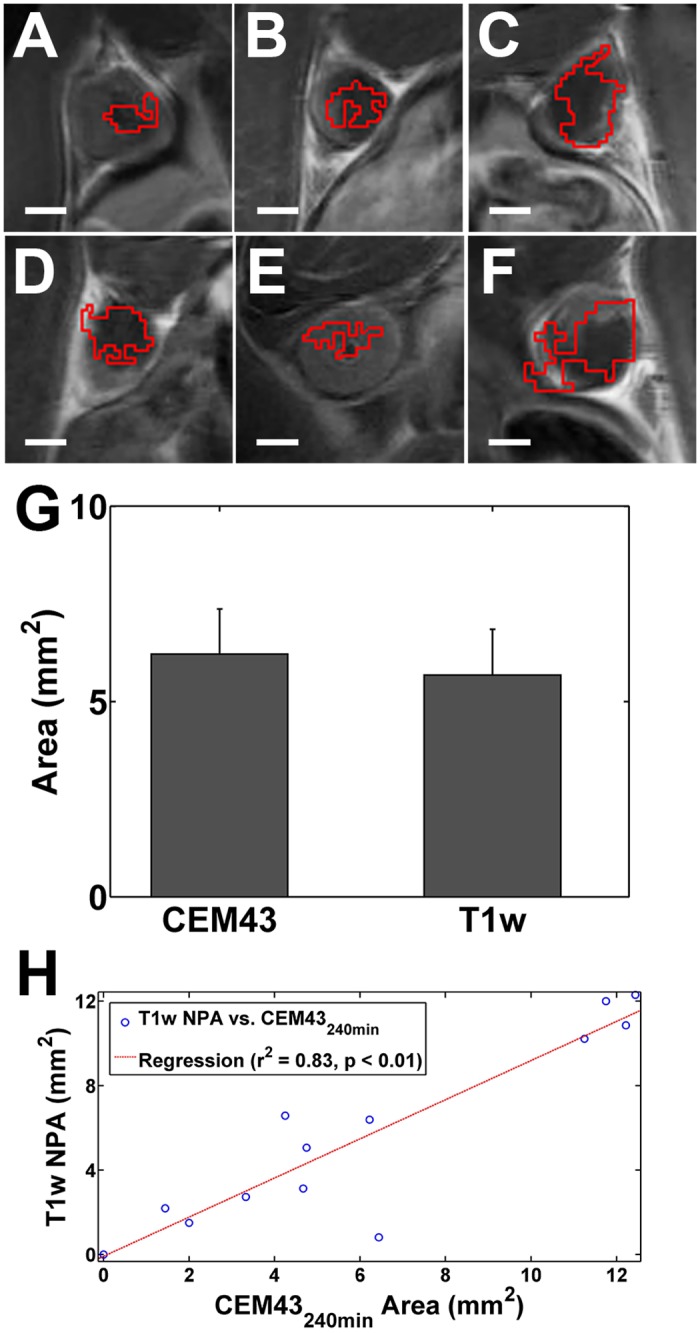
NPA compared to thermal dose. (A-F) Post contrast T1w images immediately following thermal ablation of 6 individual animals. The red contour approximates the region of CEM_43_ = 240 min. The under perfused regions are visualized as dark regions within the tumor. Scale bar represents 2 mm. (G) Immediately following ablation the area of thermal dose CEM_43_ = 240 min is not significantly different than the under perfused region across the n = 13 animals examined at this time point, and (H) the two appear correlated. Error bars represent ± SEM.

The contour of CEM_43_ ≥ 240 min was overlaid on the post contrast T1w image (Fig. 1A-F) for visual comparison with the NPA. The NPA measured on the focal plane of the ultrasound was 5.7 ± 1.2 mm^2^, while the area of tissue receiving a CEM_43_ ≥ 240 min on the same plane was 6.2 ± 1.2 mm^2^ (Fig. 1G). The NPA and area of thermal dose CEM_43_ ≥ 240 min were found to be correlated (r^2^ = 0.83, p < 0.01) and not significantly different (Fig. 1H).

**Fig 2:**
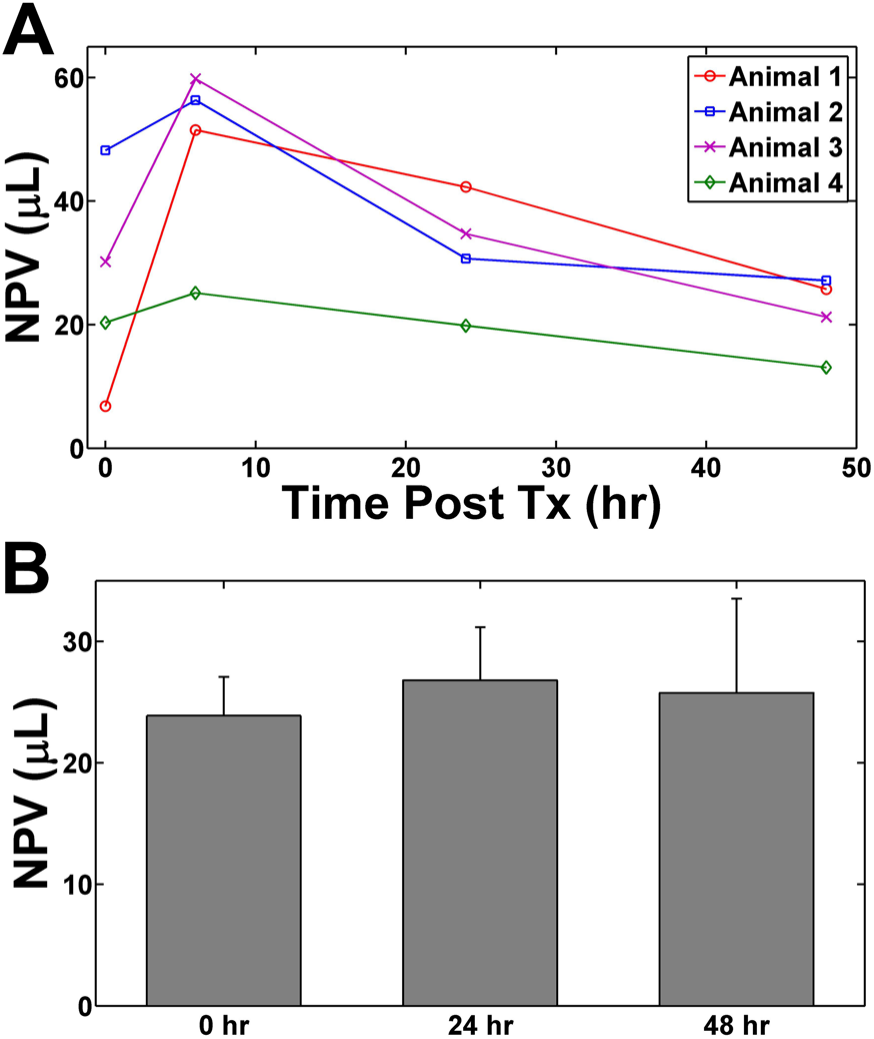
The progression of the NPV over 48 hours. (A) The NPV of 4 individual animals at 0, 6, 24, and 48 hours post ultrasound ablation. (B) The mean NPV of all single spot ablation animals at 0, 24, and 48 hr post HIFU ablation. Although there appears to be a large increase in the NPV post treatment (between 6–8 hours), the NPV at 48 hours is not significantly different from the immediate post treatment results suggesting that regions not well perfused on contrast enhanced T1w imaging may be indicative of ultimate tissue damage. Error bars represent ± SEM.

We found the NPV immediately following ablation (0 hr) to be similar to the NPV at 24 and 48 hours post ablation (Fig. 2A). However, we did notice a transient increase in the NPV at 6 hours post ablation. The NPV could not be compared to the thermal dose since real time temperature monitoring (performed with magnetic resonance thermometry (MRT), via the proton resonance frequency shift method), was only performed on a single slice at the focus of the ultrasound during the ablation to increase temporal resolution. However, in the ultrasound focal plane, we found no significant difference between the NPV immediately following HIFU ablation and at 24 and 48 hours following treatment (Fig. 2B).

**Fig 3:**
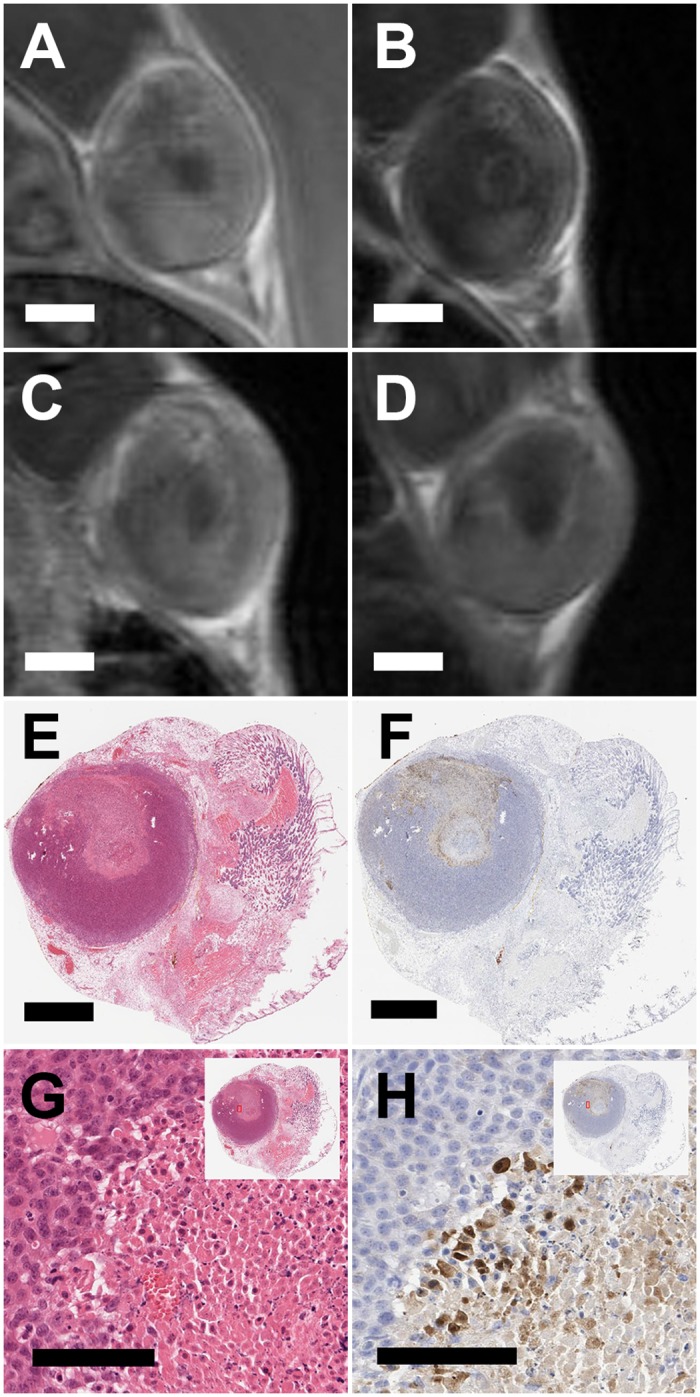
Progression of the under perfused region, seen as a dark spot or region within the tumor, within the same tumor (A) immediately following HIFU ablation and (B) 6hr, (C) 24hr, and (D) 48hr post. Corresponding (E) H&E and (F) caspase 3 along with higher magnifications of the edge of the affected region for both (G) H&E and(H) caspase 3. Scale bar in (A-F) represents 2 mm and in (G) and (H) represents 100 μm. All panes present images of the same animal, which was part of study 2 where we imaged the same individual animal at 0hr, 6hr, 24hr, and 48hr post ablation. Histological sections were obtained after the 48 hour imaging time point and histological sections were registered with the 48hr MR images by using the skin (which was left on the tumor) as a reference point.

In the ultrasound focal plane, the focal spot of the beam was consistently visible as a non-enhancing region at all time points following HIFU (Fig. 3A-D). However, the tissue surrounding the focal spot varied in its degree of enhancement over the 48 hour period.

**Fig 4:**
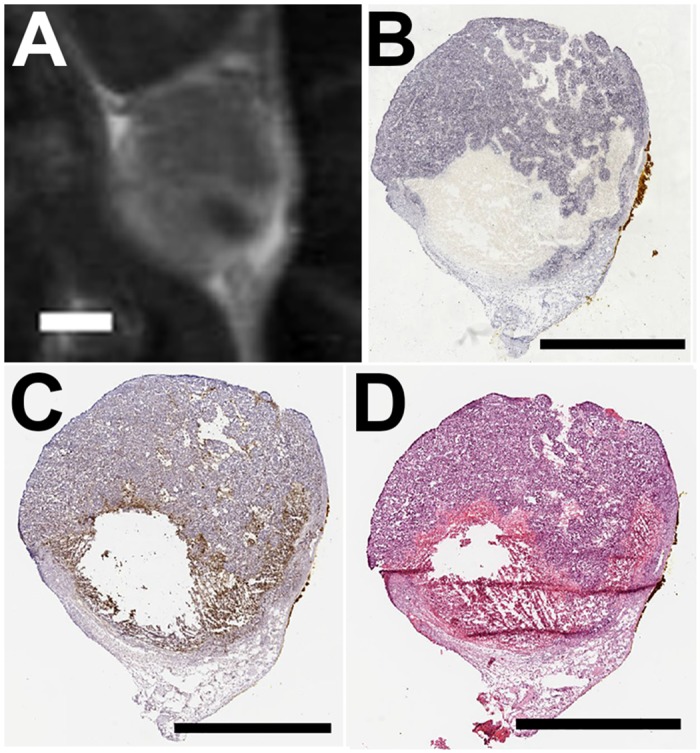
T1w contrast enhanced image at 48 hours post ablation and corresponding serial cryosections. (A) Contrast enhanced T1w image of a tumor 48 hours following HIFU ablation. The under perfused region is visible as the dark spot in the lower right corner of the tumor. Serial sections were stained for (B) NADH diaphorse, (C) caspase 3, and (D) H&E. Scale bar represents 2 mm in each case. All panes present images of the same animal. Histological sections were acquired immediately following 48 hour MR imaging (A) and were registered with the image by using the skin interface as a reference point.

Morphologically, the NPA, as visualized on the focal plane of the ultrasound at the 48 hour time point (Fig. 3D), was similar to the region of tissue death observed on the H&E images (Fig. 3E) (animal was sacrificed immediately following 48 hour MR imaging to acquire histology). The higher magnification H&E image shows a sharp demarcation of viable and non-viable cells (Fig. 3G). A serial section stained for caspase 3 (Fig. 3F) shows a regionally extensive area of necrosis in the ablated region, and a rim of apoptotic cells (Fig. 3H) separating the viable tissue from the necrotic core. Additionally, with serial cryosections we found the non-perfused regions on the contrast enhanced T1w images (Fig. 4A) to be morphologically similar to the non-viable regions on histology, and non-viable tissue regions on NADH diaphorase (Fig. 4B) aligned with regions expressing caspase 3 (Fig. 4C) that were non-viable by H&E (Fig. 4D).

**Fig 5:**
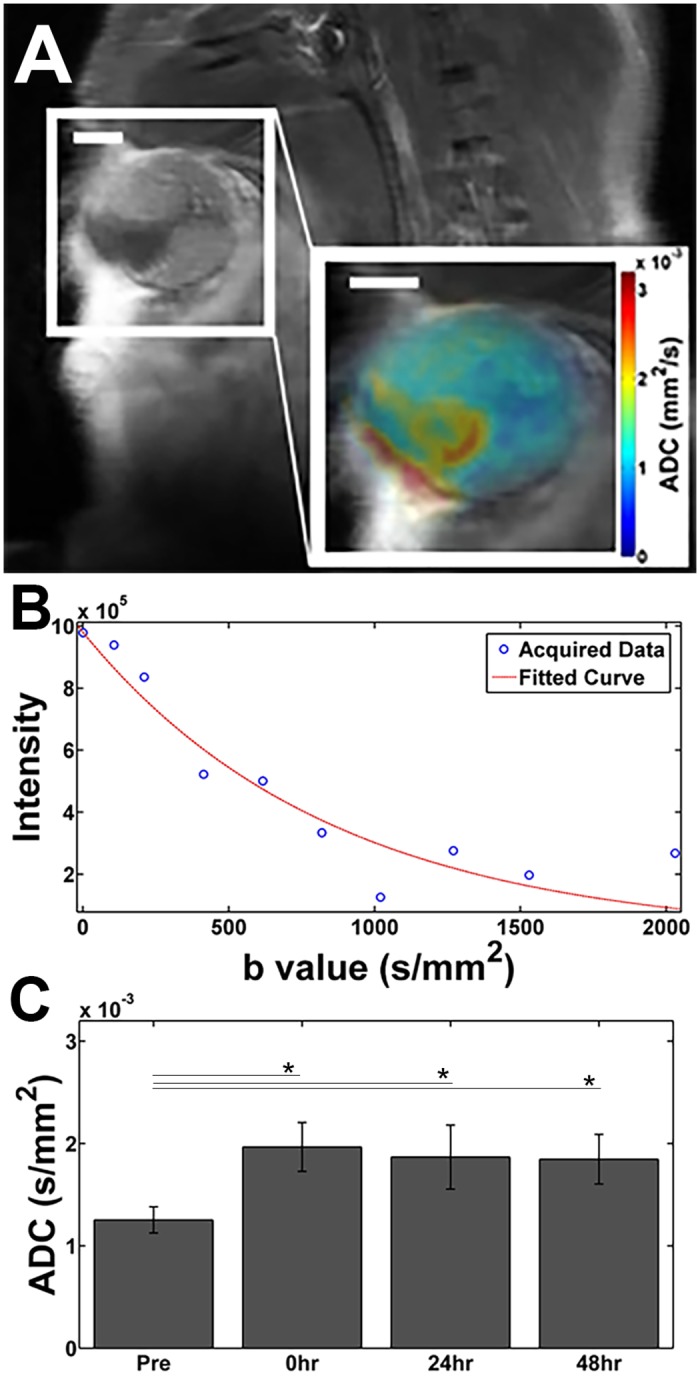
Contrast enhanced T1w image at (A) 48 hours post thermal ablation, inset is a close up of tumor with ADC map overlaid in color. Using the entire tumor from (A) as an ROI, we get (B) an average ADC for the tumor by fitting to a mono-exponential. Scale bar represents 2 mm. (C) Tumor ADC (mean over all animals). *: p < 0.05.

We observed heterogeneity in the ADC maps of the tumors following HIFU ablation (Fig. 5A). Using an ROI of the whole tumor fit to a monoexponential curve (Fig. 5B), we found the ADC of the whole tumor increased immediately following HIFU ablation (Fig. 5C) and remained significantly increased over pre-ablation values through 48 hours.

**Fig 6:**
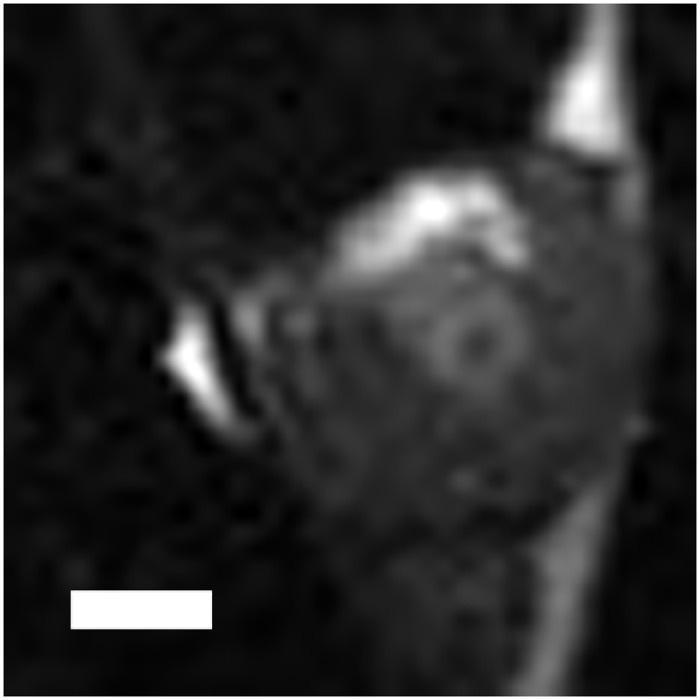
T2w image of a tumor 24 hours following HIFU ablation exhibiting a hyperintense rim around the ablated spot suggesting edema. Scale bar represents 2 mm.

**Fig 7:**
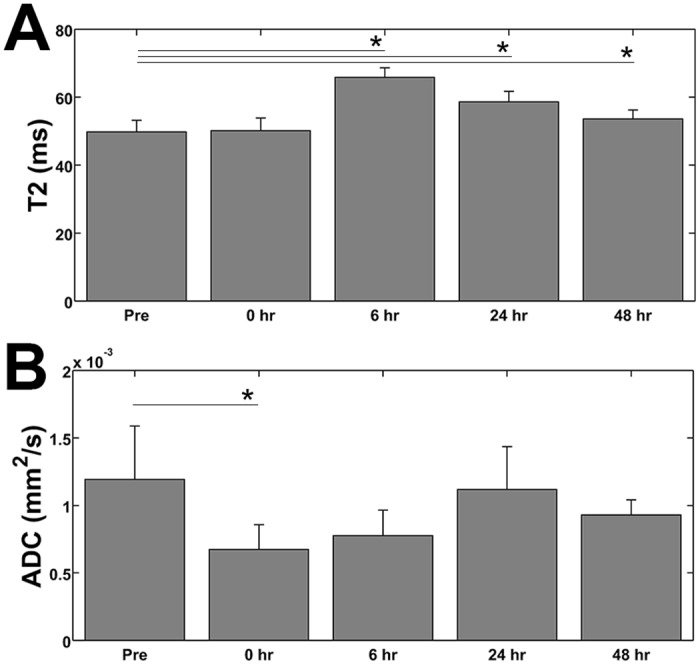
T2 and ADC values of the ablated region. (A) The measured T2 values of the ablated region within the tumor illustrate an increase in T2 starting at 6 hours post treatment and remaining elevated, with respect to pre-ablation values, through 48 hours. (B) The change in the ADC at the ablated region/spot of the tumor shows a decrease immediately following treatment. The ADC remains slightly elevated through 48 hours, but we only found the decrease to be significant (compared to the pre-ablation values) at the 0 hr time point. *: p < 0.05.

We found no significant difference in tumor T2 (using an ROI over the entirety of the tumor) at any of the time points. However, similar to the ADC maps, we observed heterogeneity on the T2 maps. On the T2 weighted scans of some animals, we observed a hyperintense ring surrounding the inner non-perfused region suggesting edema (Fig. 6). When drawing the ROI solely around the ablated region, we noted a small, but significant, increase in T2 at 6, 24, and 48 hours post ablation (compared to pre-ablation values), but no change in T2 was noted immediately following ablation (Fig. 7A).

Similarly, when we focused solely on the ablated region on the ADC maps, we found that the ablated region exhibits a significant decrease in the ADC immediately following ablation (Fig. 7B). Moreover, the ADC remains decreased at the focal point of the ultrasound beam throughout all the time points examined. However, the decrease in ADC was found not to be significant (relative to the pre-ablation values) with the exception of the 0 hr (immediately following ablation) time point.

## Discussion

The region of tissue death following HIFU ablation was best visualized on contrast enhanced T1w images as hypointense regions, namely regions that did not enhance following contrast administration. Interestingly, although we observed an increase in the NPV at the 6 hour time point, we did not find a significant difference between the NPV immediately following ablation and the 24 and 48 hour time points. This suggests that the final extent of tissue damage following ultrasound ablation can be predicted from immediate post ablation contrast-enhanced T1w scans. However, in our study, we focused primarily on single spot ablation, which inherently has short treatment times. It is possible that a longer treatment (e.g. attempting to achieve full coverage of the tumor) may allow the NPV to grow (as we observed at the 6 hour time point) and cause an overestimation of tissue death.

We found agreement between the NPA and the area of tissue receiving a thermal dose of at least CEM_43_ = 240 min, which suggests that the thermal dose maps generated during an ablation procedure are also of great utility in predicting final tissue death.

A sharp demarcation between the viable and non-viable cells was observed on H&E, NADH diaphorase, and caspase 3 stained sections by 48 hours post ablation. The sharp boundary observed on histology indicates HIFU to be capable of high precision thermal dose delivery but also underscores the need for accurate determination of treated tissue to ensure full and adequate coverage of the target region. That is, the great precision of thermal dose delivery also makes it easier to potentially, inadvertently, leave small “islands” of tumor untreated. Moreover, the similarity between the regions of viable and non-viable tissue on NADH diaphorase and H&E stained sections at the later time points further suggests that H&E is adequate in identifying HIFU ablated tissue. However, given the desire to immediately assess treatment efficacy in some therapies, NADH diaphorase imaging may be required under some circumstances.

Although T2w imaging is useful in identifying ablated regions in several tissue types, we found the region of ablated tissue to be difficult to discern, which is consistent with previous studies which have indicated that tumor tissue shows limited changes in T2 following thermal ablation [42].

The elevation of the whole tumor ADC indicates enhanced diffusion of water molecules results from the partial ablation procedure. Following thermal coagulation, it would be expected that diffusion would be more restrictive as proteins are denatured. In the region of ablation, this may be expected, however, considering the tumor as a whole, most of which was not ablated in this study, an increase in perfusion and vascular leakiness is expected in response to thermal injury, which is consistent with the observed increase in tumor ADC. Moreover, in the region of the ultrasound focus, we observed a decrease in the ADC following HIFU ablation, which is consistent with localized thermal coagulation.

We recognize some limitations of our current study. Namely, we did not explore dynamic contrast enhanced (DCE) MRI in the current study, which would likely prove more sensitive to changes in tumor perfusion. Future studies would evaluate the utility of DCE-MRI, and the calculation of permeability coefficients, as another possible predictor of tissue death. The inclusion of parametric K^trans^ map is a goal for a future study. Additionally, we examined a small number intermediate timepoints between 0 hr and 24 hr. Including additional timepoints such as 1, 3, 12, and 18 hours post ablation could improve our understanding of physiological changes. Furthermore, we primarily examined single spot ablation, but future studies may focus on complete thermal ablation using MRI to identify remaining viable tumor.

## Conclusions

Of the protocols examined in this study, contrast enhanced T1w imaging offers the best delineation of ablated tissue. The NPA on contrast enhanced T1w images correlates with the lethal contours (CEM43 ≥ 240 min) of thermal dose maps. Furthermore, we found that the NPA immediately following ablation provides a good estimate for the final degree of tissue death as observed in later histological sections. For immediate histological assessment of the ablated volume, NADH diaphorase or a similar protocol is required. While we did not find changes in T2 and ADC values following HIFU to be as sensitive as contrast enhanced T1w imaging for identifying ablated tissue, these protocols are promising and deserve further investigation.

## Supporting Information

S1 Fig A full resolution scan of the histological section shown in Fig. 3E. Scale bar represents 2mm.
(TIF)

S2 Fig A full resolution scan of the histological section shown in Fig. 3F. Scale bar represents 2 mm.
(TIF)

S1 Video A series of temperature maps overlaid on T1w images during thermal ablation. Thermal maps were generated in real time during the ablation procedure at a temporal resolution of 0.5 Hz.
(MP4)
