# Supplementary figures and images for: Magnetic Resonance Imaging Assessment of Effective Ablated Volume following High Intensity Focused Ultrasound
Source: PLoS One. 2015 Mar 18;10(3):e0120037. doi: 10.1371/journal.pone.0120037 (PMC4365027; doi:10.1371/journal.pone.0120037)

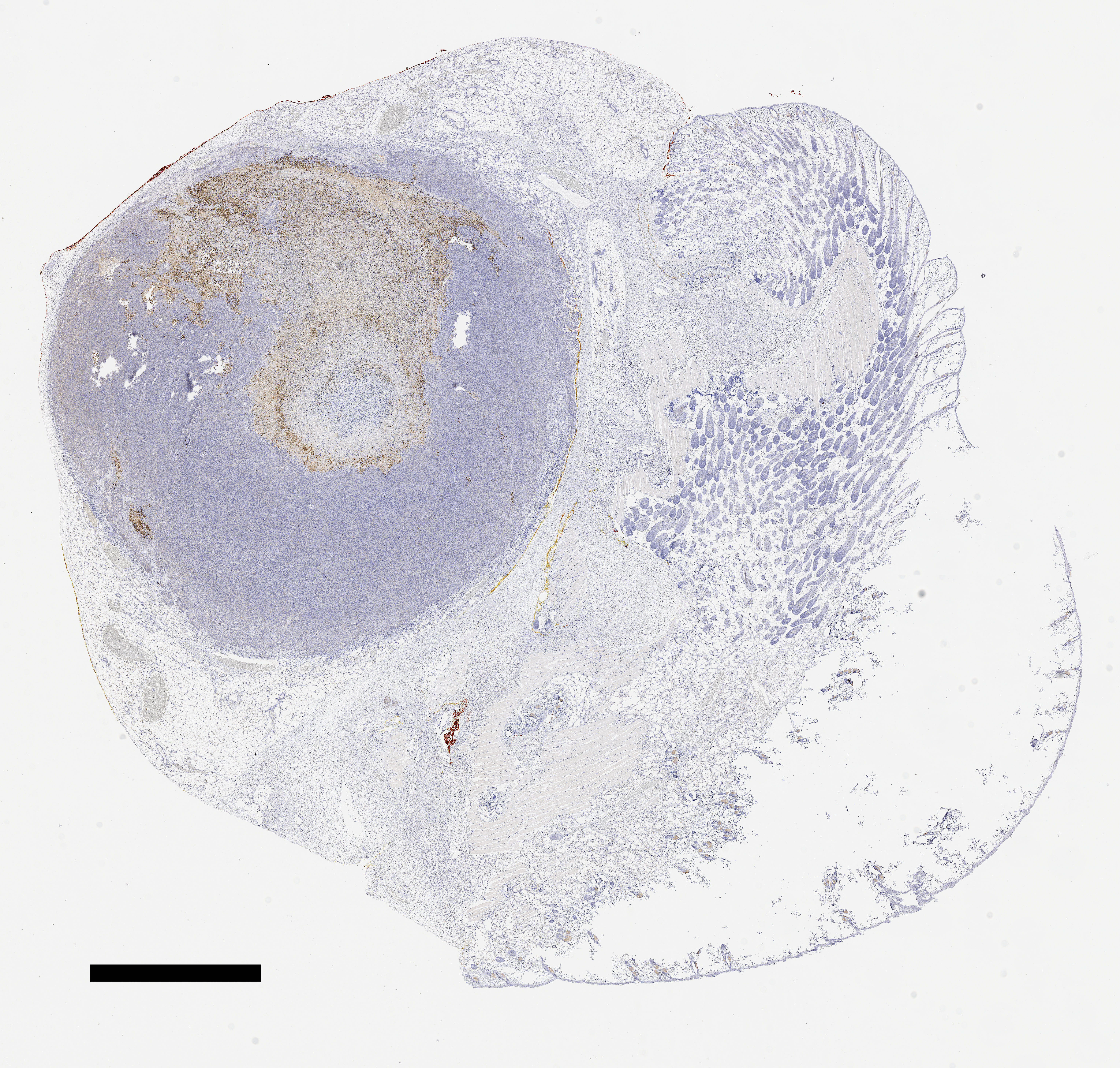

Supplement: S2 Fig — Scale bar represents 2 mm. (TIF) [file pone.0120037.s002.tif]
